# Biomolecular Prospecting, Informative Gaps, and the Cancer Clinic: A Qualitative Fieldwork and an Epistemological, Historical and Ethical Analysis of Informed Consent for Clinical Trials for Monoclonal Antibodies and Biobank Research

**DOI:** 10.3389/fgene.2022.872211

**Published:** 2022-06-13

**Authors:** Flavio D’Abramo, Annemieke Bont, Lisa Nüßlein

**Affiliations:** ^1^ Charité Comprehensive Cancer Center, Berlin, Germany; ^2^ Division of Health Psychology, Free University of Berlin, Berlin, Germany; ^3^ Max Planck Institute for the History of Science, Berlin, Germany; ^4^ Independent Researcher, Berlin, Germany; ^5^ Institute of Social and Cultural Anthropology, Department of Political and Social Sciences, Free University of Berlin, Berlin, Germany

**Keywords:** monoclonal antibodies, clinical trials as topic, biological specimen banks, databases as topic, informed consent, qualitative research, history of medicine, ethics

## Abstract

What happens to patients with cancer engaged in biomedical research when intellectual property regimes and ethical regimes intersect? This qualitative historical study addresses this question by situating the experiences, hopes, and reasons of patients to enter clinical trials within the historical trajectory of informed consent and monoclonal antibodies, the biotechnology underpinning many targeted drugs used in oncological clinical trials and biobank research. Based on fieldwork we undertook in a German university hospital where we interviewed patients and the medical personnel, a historical review, and an ethical analysis we inquire into the effects that financial, legal, and technological changes connected to the relevant pharmaceutical research and commerce have on cancer patients engaged in clinical trials and biobank research. We find that the controversial aspects of monoclonal antibodies, especially those related to the commercial interests at stake, enter the informed consent process mainly in the form of informative gaps. We highlight how a qualitative analysis of the clinic, especially when it is situated against the backdrop of the history of related technological advancements and patent regime, it can serve the purpose of giving voice to subjects who are silenced by regimes of an ethical, epistemic, and commercial kind while pointing to informed consent as an unhelpful device for addressing risks arising from the commercial purposes of biomedical products and infrastructure.

## Introduction

In this study, which combines an empirical ethics research with a historical analysis, we have thought extensively about the effects of the entrance of monoclonal antibodies in the clinic, in the form of experimental drugs for cancer patients^1^ Likewise, we have reflected on what happens when information on monoclonal antibodies used as oncologic targeted drugs is conveyed to patients *via* informed consent, and how patented monoclonal antibodies change what it means to be a patient. These questions intrinsically relate to informed consent as an interface between science and society (D'Abramo, 2015), especially when experimental scientific products are clinically administered to research subjects, thus producing expectations, hopes, concerns, and a whole new set of relationships between all the actors directly engaged in pharmaceutical trials—i.e., patients, patients’ loved ones, nurses, doctors, and researchers.^2^ Some of the concerns we had in mind and that structured our fieldwork in 2015-2016 have endured till the time of writing in 2019-2021, especially those at the crossroads of history of science and qualitative research. We focused therefore on informed consent, which provides ethical authorization for companies and research institutions to engage with both patients for their bodily data, and medical personnel for their work and human communicative abilities—i.e., translating the technical language of clinical trials and biobank research of informative sheets used to obtain informed consent into terms that patients could understand. We conceive here of the more practical aspects of ethics regime as a set of activities implemented by the medical personnel that make possible both biobank research and the provision of materials on which biobank research is based and from which new drugs are produced, tested, approved, and marketed. The ethical regime through which biobank research is governed allows the very extraction of elements necessary for drug production and drug testing, which are the biological samples of patients and their personal data^3^ prospected as possible resources to produce biomolecular therapeutics for so-called personalized or precision medicine. Indeed, in the century-long development of cell culturing, the key concepts and related practices of the *immortalization of cell lines* and their *commercialization* as biomedical products have relied on the *individual information* needed to make the analogy between cells and whole organisms, especially within the biotechnological sector of monoclonal antibodies, that thrives on immortalized cell lines (Landecker, 2007). Heavily processed data generated by clinical trials, of which biobank research is a part, are “one of the key ingredients of a drug, crucial to bringing it to the market and to making it circulate in that market” (Sismondo, 2018: 41).

This study emerges from a qualitative fieldwork that we undertook in a German university hospital to analyze patients’ understanding of informed consent sheets to enter oncologic clinical trials and biobank research as well as to scrutinize expectations, concerns and more general experiences of patients and the medical personnel. In the qualitative phase we utilized two questionnaires, interviews with patients, focus groups with the medical personnel, and we coded the conversations we had with patients. The fieldwork we undertook motivated us to develop a critique of the ethical regimes and standards used in pharmaceutical clinical trials, especially when by adopting these standards patients’ voices are lost. Later, we developed an analytical framework to understand the historical codetermination of biomedical research, health care systems, politics, and economy at both national and international scales.

We intend to make the voices and experiences of patients and the medical personnel visible so as to contribute to the debate on the role that social (or non-epistemic) values have and could have in medical research and to support the care of these patients. Although the objective knowledge of biomedicine and other sciences is associated with neutrality and objectivity, its scaffolding includes social, economic, political, and subjective factors (Longino, 1990; Harding, 1992; Douglas, 2009; Kourany, 2010; Longino, 2018) that are at the heart of many health issues (Lock and Nguyen, 2018). Patients’ lack of understanding about experimental procedures distances medicine from democracy, in the sense of the possibility of access to information about the regulatory, economic, and technoscientific workings of biomedical procedures (transparency), and in the sense of a science in which patients assert their values—for example, the aspirational self-determination of individuals and social values such as access to and distribution of medical services and products. Indeed, patients’ social values do not necessarily coincide with the epistemic values of biomedical research. Nevertheless, some medical interventions for patients with advanced cancer use their firsthand experiences to develop effective psychosocial interventions (D’Abramo et al., 2016), which is a reason to include patients’ voices as epistemic element that ameliorates clinical outcomes. Last but not least, patients’ lack of understanding also translates into the hope developed by patients at the end of life and their loved ones that experimental drugs may have curative effects so as to overshadow the difficult and sad process of the final goodbye (D’Abramo and Guastadisegni, 2012).

Our aim is therefore to show that ethical standards for biomedical research are the result of social negotiations, and that epistemic standards, for instance those used to handle clinical trials, are the result of social encounters among actors whose values, material conditions, and experiences might widely diverge—or who simply have a hard time speaking to each other (Sunder Rajan, 2006; Petryna, 2009; Sunder Rajan, 2017). Critical approaches to informed consent and bioethics are far from being new, for instance there are plenty of studies on patients’ lack of understanding of informed consent for clinical trials (cfr. D'Abramo et al., 2015). Nevertheless, our study represents originality in its multidisciplinary approach—i.e., applied ethics/qualitative research and the history of science—on informed consent for clinical trials on monoclonal antibodies and biobank research.

In the next section we introduce the fieldwork undertaken at the Charité University Hospital in Berlin, where we administered two questionnaires to cancer patients engaged in clinical trials and biobank research. In the second section, we review the results of the fieldwork, where the patients expressed their lack of understanding of information contained in informative sheets. In section three, we review the history of immortalized cell lines and monoclonal antibodies, biobank research and the patent and ethical regimes through which they are regulated, and the effects that these regimes have on the possibility of providing adequate information to patients. In section four, we review the effects that the ethical and commercial regimes within which targeted drugs are developed and tested have on the expectations of cancer patients and their loved ones, to finally highlight the limitations of informed consent for risks derived from the commercialization of biotechnology products derived from biological samples and data of patients.

## The Fieldwork

Our fieldwork was motivated by our wish to critically disentangle the constraints on the communication between patients, the medical personnel, and medical institutions in the context of pharmaceutical research on monoclonal antibodies, specifically by looking at the ethical regimes to handle the experimental practices. To operationalize the research, we adapted a questionnaire developed by Ormond and colleagues (Ormond et al., 2009) to interview patients at both the oncology wards and the oncological stationary unit of Charité Comprehensive Cancer Center at the Benjamin Franklin hospital in Berlin. We discussed the questionnaire with oncologists and we fined—tuned the questions through three pilot interviews so as to facilitate patients’ understanding. The questionnaire A focused on patients’ understanding of information on the clinical trial and how they decided on whether to enter medical research (Supplementary Questionnaire A—Table S1). From July 2015 till March 2016, we selected (together with the medical personnel) both inpatients and outpatients. We sought a balance between genders and the representation of at least three generations, meanwhile asking the medical personnel whom they considered in a good enough medical condition to sustain an interview. After having received the completed questionnaire from the patients, we asked them to do an interview to review their responses in order to allow them time and space to address the issues that they felt were important in their specific situations. Most of the trials in which the interviewees were part of, were designed for the approval of targeted drugs based on monoclonal antibodies. The outpatients were interviewed at the ambulatory unit, where they were on friendly and informal terms with the study nurses, whereas our interactions with the inpatients took place in the oncology wards, where the medical personnel facilitated comfortable encounters in sometimes critical contexts—i.e., patients suffering for the disease or for medicaments’ side effects. Besides the interviews with patients, we had conversations with medical personnel and researchers, and focus groups with nurses, biologists, physicians, and psycho-oncologists. Moreover, we considered the broad consent forms given to patients to authorize the use of their biological samples and clinical records. We therefore administered a questionnaire on biobanking (Supplementary Questionnaire B—Table S2). We asked all the patients for their written consent to participate in our study, which was authorized by the hospital’s ethics committee. After having transcribed the interviews, the three of us coded them by using Atlas.ti and through codes that we developed deductively, from the literature, and inductively, from three pilot interviews and after extensive discussion (Supplementary List Of Codes—Table S3). In turn, we also added additional codes during the analysis of the text that we ultimately validated. For all codes we developed inductively, we referred to grounded theory methodologies. We decided not to quote patients’ interviews for privacy reasons.

## Results of the Fieldwork

We asked thirty-one patients to participate in our study (*n* = 31, response rate 30/31, nineteen-seven percent). The questionnaire revealed that the average age of patients was 60.43 years old. About one half of patients had a diagnosis for metastatic, *non-curable* cancer—these patients were engaged in trials where standard therapy was combined with experimental drugs. The other half of patients had a *non-treatable* disease for which no standard drugs to stop cancer progression were available—these patients were engaged in trials where they received only experimental medicaments. Twenty-six patients filled out the questionnaire A, and we interviewed twenty-six of them (the average time of interviews was 28 minutes). The questionnaire on biobanking (Supplementary Questionnaire SB) was filled out by twenty-two patients, seven of whom were interviewed (the average time of interviews was of 8 minutes). Twenty-four interviews were audio recorded (Supplementary Questionnaire SA), transcribed verbatim, translated from German to English, and analyzed, while for two of them we took hand notes. We analyzed both the filled questionnaires as well as the interviews to distillate our results. Ten out of twenty-six patients found the information they received on the clinical trial (Supplementary Questionnaire SA and interviews), contained in the information sheets, to be barely understandable (thirty-eight percent); nine of them found the information sheets partially understandable (thirty-five percent); three of them found the information fully understandable (twelve percent); while ten of them did not remember or did not answer the question for other reasons. Twenty patients told us during the interview that followed questionnaire A, to have received information on the clinical trials from the medical personnel (seventy-seven percent). Nine of the twenty-six patients interviewed following their answers to questionnaire A, decided to participate in the clinical trial because they did not have other options (thirty-five percent), eight of them participated for altruistic reasons such as to help produce drugs for the next generations (thirty percent), and ten conceived of their participation as a good opportunity, for instance to speed up diagnostic procedures, or believed that their participation in the clinical trial would have curative benefits (thirty eight percent). Moreover, two of them stated that they had entered the clinical trial to please family members (eight percent).

What we witnessed during our interviews, was a concern expressed by patients and the medical personnel about a health care system with values that differ from their own. In particular, the symbolic meanings that oncological patients attribute to signing consent forms often vary from the broader meanings attributed to these forms by medical institutions. Whereas clinical trials are *de facto* non-therapeutic, patients frequently interpret them as therapeutic interventions. For one, information on the non-therapeutic nature of the trials is not understood (or not clearly enough); moreover, the boundaries between the therapeutic procedures experienced by patients are often blurred with the provision of experimental drugs. The effect is that, for patients, signing an authorization might symbolize an increased probability of recovery, despite the real aims, risks, and benefits of the clinical trials. Medical institutions, on the other hand, confer an overwhelming (but divergent) symbolic value on consent, which, as a psycho-oncologist told us, translates into endless consent forms for patients to sign and thus an information overload (Psycho-oncologist, 2015).

In the case of our fieldwork, the failure of informed consent to inform subjects of research regards not so much the impossibility of foreseeing future directions of research, as is commonly stated in the bioethics literature (Boniolo et al., 2012; Hansson and Levin, 2003), but rather the information overload to which the patients we interviewed were constantly subjected. When we asked patients for their opinion on a dynamic consent process based on electronic procedures that would update them on the research using their samples and data and offer the choice to renew their consent for each new project (Kaye et al., 2015),^4^ they expressed doubts related to the further burden all this information would put on them (Supplementary Questionnaire SB and interviews). At the same time, they expressed skepticism as to the integrity of the information they received: many of the patients with whom we interacted were aware of the secrecy maintained by medical institutions around biological samples and personal data, and as a consequence were deeply resigned (Supplementary Questionnaire SB and interviews).

Even if very few patients among those interviewed had gained an understanding of the clinical trial and biobank research via informed consent forms, nurses and doctors were of invaluable help in informing patients of the logistical and technical details of the clinical trials (Supplementary Questionnaire SA,B and interviews). Especially the nurses, the psycho-oncologists, and specialist doctors (*Fachärzte*) tended to all the patients’ needs. These same medical personnel navigated through the uneasy conditions of the clinic, including lengthy shifts, a rising ratio of patients per worker, and precarious contractual conditions (Cooper and Waldby, 2014). Although the ethical regime behind clinical trials translated into an overload of incomprehensible information, the cooperation between the medical personnel and informed patients—especially the patients who understood the non-therapeutic nature of all experimental drugs—resulted in some positive clinical outcomes, especially when subjects of research aware of the experimental nature of the drug on trial can offset their participation through a timely access to diagnostics and a better care (Supplementary Questionnaire SA and interviews).

On the one hand, it is helpful to understand the values of patients by focusing, through dialogues, on their histories, expectations, and concerns. But it is likewise helpful to understand the material and historical backgrounds of biotechnological and infrastructural changes, and their underpinning values. Even though as we show below the scientific literature on monoclonal antibodies is rich in details on their historical, social, clinical, economic, and technical characteristics, in all the information sheets provided by the manufacturers that we analyzed, we found basic information about this technology very rarely, if ever. Based on the lack of understanding of the information contained in the information sheets that the patients we interviewed showed, and the vagueness of the information contained in these documents, we decided to review the historical literature on monoclonal antibodies, biobank research and informed consent, in order to frame the vagueness of the information sheets and patients’ lack of understanding against the backdrop of the knowledge developed about key aspects of these elements.

## The History of Monoclonal Antibodies and Biobank Research Amidst Innovation, Legal Controversies, Patents, and Informed Consent

Despite in the early twentieth century, scientists such as Alexis Carrel tried to culture cells of non-human animals, such as the beating cells of the chicken heart, with the aim of making them immortal, mostly by cyclically changing their substance of culture (Landecker, 2007), cells outside the body were firstly immortalized in 1951, when cancer cells were extracted from the body of Henrietta Lacks (Brown and Henderson, 1983). The HeLa cells, a discovery that allowed for successful global undertakings of biomedicine such as the production of the polio vaccine and the eradication of poliomyelitis, were celebrated for decades. In the 1993 “Witness Seminar,” historian of science Robert Bud noted that monoclonal antibodies were one of the three most important biotechnological discoveries of the last century, producing a vast medical, commercial, and industrial impact (the other two being recombinant DNA and new fermentation techniques) (Robert Bud in Tansey and Catterall, 2008).^5^ Monoclonal antibodies are made out of immortalized human cancer cells fused with mouse cells immunized to produce specific antibodies. The various antibodies produced by such cell cultures can selectively bind to antigens, thereby signaling the presence of specific microbes usually associated with certain diseases or immune responses (hence their use in many antigen tests). In oncology, monoclonal antibodies can ligate with specific receptors of cancer cells so as to inhibit specific functions that typify cancer (Hanahan and Weinberg, 2000; Hanahan and Weinberg, 2011). Monoclonal antibodies, produced out of hybridoma technology, which are nowadays essential to diagnostics and therapeutics at the global scale, were “discovered”^6^ in 1975 by British scientists César Milstein and Georges Köhler, whose milestone paper highlighted the commercial and industrial implications of their discovery (Cambrosio and Keating, 1988). Three years later, the first patent on monoclonal antibodies was granted to researchers of the Wistar Institute in Philadelphia for a “Method of producing [an anti-influenza] antibody” derived from the cell line of Milstein and Köhler (European Patent Office, 2021). The patent office of the UK and other states rejected the patent that the US office granted to Wistar’s researchers, a dispute that since monoclonal antibodies’ inception characterized them as biotechnology able to elicit national and international disputes (Mackenzie et al., 1990; Turner, 2012). The hybridoma technology raised legal conflict also between patients and private, for-profit enterprises—i.e., health care providers. In the 1990s, the HeLa cells became an emblem of the commercialization of body parts without the donors’ knowledge. Immortalized cancer cells—able to endlessly reproduce themselves and derived proteins—represent both a medical and commercial triumph as well as one of the central themes of ethical dispute associated with technologies of such extraordinary clinical and commercial success (Beskow, 2016; Turner, 2012). Later, when John Moore, a patient with leukemia who discovered in the 1990 that the biological samples extracted from his body were being used to develop and patent a cell line and all the products derived from it, he sued for a share of the profits gained through the cell line (*Moore v. Regents of UCLA*) (Landecker, 1999).

In a 1977 meeting of the World Intellectual Property Organization—before standards for monoclonal antibodies were set—an agreement established among member states required that samples of biological material proposed for a patent be deposited in biological repositories and made freely available to anyone on demand (Mackenzie et al., 1990). The requirement to deposit samples of biotechnological inventions based on cell cultures went together with the establishment of banks of biological samples, or biobanks, starting in the early 1980s. In 1980 the American National Institute of Allergy and Infectious Diseases supported the creation of the Hybridoma Cell Bank. In 1983 the Hybridoma Data Bank was established, hosted by the American Type Culture Collection (Cambrosio and Keating, 1995). In the same period, which witnessed the exponential growth of biotechnology enterprises, Canadian, US, and European research institutes encouraged technology transfer between basic and applied scientific research (Krimsky, 1991). During the 1970s, the vast use of monoclonal antibodies and the initial lack of standards for the industrial infrastructures that produced them led to major breakdowns—e.g., the distribution of genetically contaminated monoclonal antibodies—pushing the biomedical sector to establish standards for the hybridoma technology. During the 1980s, when science started to be subsumed under technology (Forman, 2007), the World Health Organization, the United Nations Development Programme, the World Bank, and other ad-hoc scientific organizations arranged international meetings to reach a set of international agreements and related standardization procedures for the production and commerce of monoclonal antibodies, seminars, and other initiatives (Cambrosio and Keating, 1995). The establishment of the first biological repositories for cell cultures went together with the lively activity of UN agencies coordinated by the World Bank so as to concretize a globalized development economy guaranteeing a specific world order (Staples, 2006).

**FIGURE 1:**
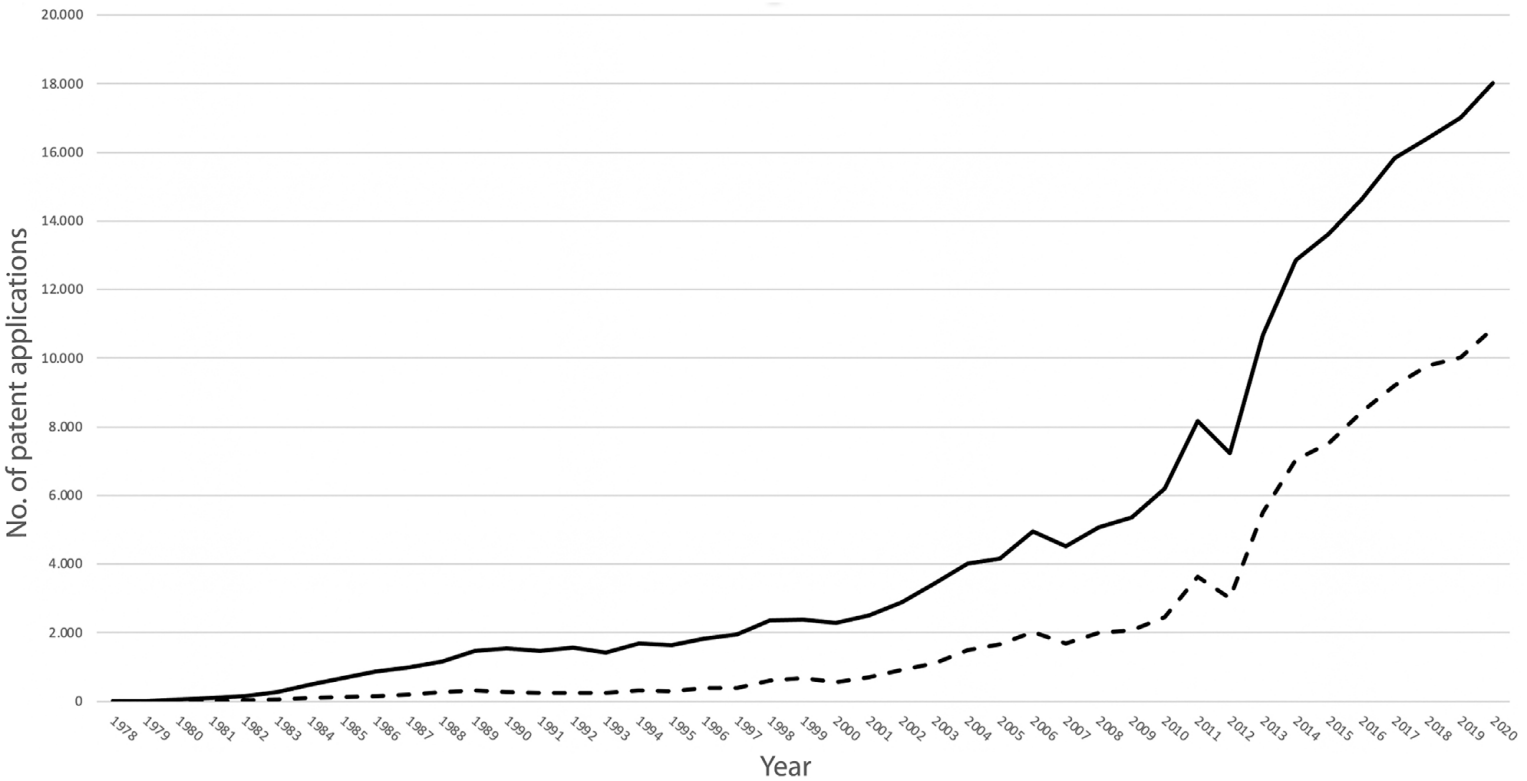
Number of patent applications for the period 1978-2020 for inventions based on monoclonal antibodies (continuous line) and for inventions based on monoclonal antibodies intended for the oncology sector (dotted line) (Graph based on data of the European Patent Office, 2021).

The expanding production and commerce of monoclonal antibodies were mirrored in the trajectory of patent claims over the last 40 years. The patent claims for monoclonal antibodies in 1978 were nine in total. By 1990 the number of claims had increased to 1.528, reaching 6.195 in 2010 and skyrocketing to 18.028 in the year 2020 (European Patent Office, 2021). Out of the applications for patents regarding monoclonal antibodies filed in 2020, more than ten thousand (*n* = 10.019) were relevant to cancer research, cancer diagnostics, or cancer therapeutics (Figure 1).

The high level of patent litigation that characterizes the production and use of monoclonal antibodies since their inception in the late 1970s, and other biotechnological products (Mirowski, 2008), has had an impact on the free, non-proprietary scientific information on all related biomedical products.^7^ Mackenzie and colleagues have shown that when used in the arbitration context, the role of free scientific information primarily aims to support the appropriation of knowledge, with the effect that scientists who want to protect their ideas from being patented withhold their ideas from the public domain until fully formed (Mackenzie et al., 1990). The first litigation cases to arbitrate the ownership of specific “discoveries” based on monoclonal antibodies implied that commercial pressures defined the scientific practice itself, for instance in the “rigor” required of scientists in documenting their procedures to eventually show, during the litigation process, the paternity of ideas and of related practices (Mackenzie et al., 1990: 77). Under the current patent regime, the availability of information on monoclonal antibodies, is thus inversely proportional to their diffusion. The more widespread this technology becomes, the greater the economic interests related to this technology; the greater the commercial interests involved, the less inclination there is to share information that might be hoarded by commercial competitors. Between 1978 and 2020, the extensive commercial pressures exerted on monoclonal antibody-derived products have therefore made free scientific information less available, with effects that reach many other aspects of biomedical research, including informed consent, which rarely contains precise and comprehensible information on monoclonal antibodies tested in clinical trials, nor information on the risks involved in commercializing the information and biological samples extracted from donors.

Although biobanks have been in operation since the 1970s, informed consent started to be tentatively considered in the early 2000s as a way to authorize the participation of patients and donors in projects of research with possible non-physical risks emerging from the use (and misuse) of biological and individual data (Tutton et al., 2004; Hoeyer, 2004). The controversial aspects of sample and data collection of the Human Genome Diversity Project of the 1990s stimulated the US debate on the need, elicited by the National Science Foundation and the National Institutes of Health, to request informed consent from the donors, which in that specific case were the US indigenous communities (Lock, 2001). Unlike a clinical trial or administering a therapeutic, the risks stemming from participation in biobank research are not directly related to the possibility of physical injuries but to discrimination through the misuse of personal information. One such abuse may follow from patents and financial exploitation of biomedical technologies resulting from biobank research, and may lead to unfair distribution of drugs developed by such biomedical enterprises, or to the prioritization of profit at the expense of patient care. The risks associated with the commodification of products derived from biomedical research are well exemplified by a population living in an industrial area where exposure to pollutants causes oncological diseases. Individuals belonging to such a community might then be invited to participate in biobank research whose results lead to the production of drugs to cure the disease—drugs that might nevertheless be economically unsustainable for the people of the community who contracted the disease (Hobbs et al., 2012). Another example comes from the US, where the clinical testing of prescription drugs is conducted on uninsured individuals who then lose access to those treatments when they are made available on the market (Fisher, 2008). Since the early 1980s, the function of informed consent as a device to protect human subjects from physical injuries has been challenged therefore by global transformations elicited by the political economy and technological advancements of biomedicine and the life sciences, especially by the establishment of repositories of human biological samples and the associated patent regimes. Although today the ethical debate on the fairness of commercial interests in the medical field is a marginal aspect of the bioethical debate, the issue of patents and profit was, until the middle of the last century, a primary aspect of the codes of medical ethics—e.g., from the 1874 and till the 1912, the American Medical Association prohibited physicians to hold patents on instruments or drugs (Gabriel, 2014). Nevertheless, since 1999, when Jesse Gelsinger died as a result of a clinical trial, the US debate on the impact of financial interests of investigators onto medical conduct and the safety of patients revived (Steinbrook, 2008). And yet, unlike about a century ago, when medical associations legislated about the harmful effects of marketing medical products, today such effects are often considered, if they are considered at all, as risks that are necessarily the burden of individual patients, that is, as information to include in informed consent sheets.

Whether to consider the risks associated with the commercialization of biomedical products and services as information to be included in informed consent remains therefore an unclear point. In fact, these risks, which were previously controlled by professional associations (Gabriel, 2014), are now still under the scrutiny of governmental institutions, which, however, not infrequently prioritize patent rights to the exclusion of issues related to fair access to medical care and services, and access to correct and clear information on biomedical research.

## Discussion

The patients we interviewed did not understand information about clinical trials and biobank research for several reasons. In our fieldwork we highlighted that the lack of understanding was due to the vulnerability caused by their disease, and therefore patients showed priorities different from focusing on technical information. Moreover, we discovered that for patients with cancer engaged in clinical trials and biobank research, informative sheets and informed consent documents that the medical personnel ask them to read and sign are too many, and therefore difficult to recall. Finally, the information contained in the documents we analyzed did not explain technologies and infrastructures underpinning clinical trials and biobank research. In the historical analysis, we showed that the patent regime regulating monoclonal antibodies at the national and international levels, very likely contributes in creating commercial secrets and helps withholding information to eventually elicit informative gaps.

Despite the lack of understanding that patients showed of the informed consent documents, the motivations that brought the patients that we encountered to enter clinical trials related to an alleged common good (helping future patients) or to the alleged therapeutic agency of experimental drugs. In these cases, the ethical regime used to handle the clinical trial blurred the differences between the descriptive and the normative dimensions. Namely, the ethical regime hindered the understanding of patients so as to enable the operation of the clinical trial and biobank research. When patients (especially patients with chronic cancer who throughout years of medical care developed a deep knowledge of the clinic) entered the clinical trial and biobank research to access and benefit from better clinical facilities and services, they materially bypassed the shrinking resources of publicly funded health care. In this case, the ethical regime by which these clinical trials operate occasionally translated, thanks to the cooperation between patients and the medical personnel, into indirect positive clinical outcomes—which nonetheless helps assure the cooperation of these participants in something they arguably do not necessarily benefit from. The material value that manufacturers attribute to monoclonal antibodies therefore turns into a belief in the powers that these drugs do not (yet) have. When patients and their beloved ones trust that clinical-trial drugs will address their disease or cause a remission, their expectations are doomed to clash with the hypothetical nature of these drugs’ curative efficacy. Indeed, if the experimental drug is later found to be effective, the dosage and protocols of the clinical trial are such that the drug is in no way beneficial for the subjects of research. Therefore, clinical trials on monoclonal antibodies create both patients whose expectations will be betrayed by unkept promises as well as patients who can access better clinical facilities and services than they could otherwise afford (those partially funded by drug manufacturers).

The personal information and biological samples that patients donate need to be framed not only within the regime of hopes that cancer patients develop within their clinical and familial constellations, but also within the development of monoclonal antibodies more generally. Biological samples, personal data, and the derived molecules are indeed complementary aspects of drug production. Pharmaceutical and biotech companies developing monoclonal antibodies actually need to maintain a link between the *in vitro* cell line and the *in vivo* life, which is the pathology of the patient. As spelled out by Landecker, “the information gleaned from cells is useless unless it eventually relates back to the biology and then the pathology of the patient. Through the individual patient, the information then becomes applicable to humans in general” (Landecker, 2007: 175). Biagioli and Pottage have highlighted that personalized medicine, which the use of monoclonal antibodies in the field of oncology helps to achieve, is “a know-how whose meaning and values is conditioned by the metabolic response that it seeks to anticipate, and that takes the form of so-called information or data” (Biagioli and Pottage, 2021: 240). The personal information of donors therefore plays a key role in the bioprospecting of these samples, eventually used to develop and personalize biological medicaments.^8^ It is within this context that biological repositories used for biomedical research are complemented with information that relate to the medical records and other socioempirical information of donors, in a process in which data and samples are regarded as more important than the patients themselves (Petryna, 2009). Recent cases of the commercialization of patients’ medical records without their consent (Hodson, 2016; Lomas, 2016; Sharon, 2016) follow the same trajectory that we have described here, namely that key information about the commercial activities carried out with such medical data remain undisclosed (Nissenbaum, 2011). If the information is not disclosed, donors of biological samples and data do not have any chance to consent to their commercial use. It is on the basis of this key undisclosed information that patients’ relationships take specific shapes. However, the proposal to introduce into informed consent sheets information regarding the commercialization of biological data and samples collected from patients who are part of biomedical research remains highly controversial. The individual patient and his or her beloved ones are in conditions that hardly allow for a considered evaluation and negotiation on an equal footing. Patients with cancer, who are often with a reduced capacity of decision making, might therefore be the least subjects to benefiting for knowing such information and making decisions accordingly. In this case, respect for the autonomy of patients with reduced decision-making capacity requires that informed consent play a marginal role, for example, so as not to overwhelm them with incomprehensible information. Annemarie Mol, who has criticized the logic of choice within the medical setting, highlighted the importance of developing situations in which trust and care take precedence over self-determination (Mol, 2008). Mol’s argument is particularly relevant when the information produced and disseminated creates a burden on patients, for instance those who feel guilty because their wish not to participate in clinical trials and biobank research conflicts with the expectations of loved ones or the social contract.^9^

The approach embraced by some scientific communities in the US to have “more consent” has been indicated by Barbara A. Koenig as “the modern equivalent of a fetish” (Koenig, 2013). Similar concerns showed that an active engagement of patients participating in medical research is hardly realized through information and authorization sheets (Dawson, 2003), and that healthy or sick donors of biological samples and of personal data rarely have the chance to understand, through official documents, the characteristics, aims, risks, and benefits of research projects (Corrigan, 2003). Even if informed consent is conceived of as a device that through the disclosure of information (on the risks and benefits of medical experimentation) allows future participants to make a free and rational decision and therefore a more appropriate and informed moral judgment, research participants also “provide valuable labour and bodily material for pharmaceutical research, making them co-producers of drug products” (Corrigan, 2004: 86). It is within these political, economic, and technological transformations of biomedicine that scholars such as Kristal Biruk, Melinda Cooper, Catherine Waldby, and Margaret Lock have framed informed consent as an ethical device internal to—and not autonomous from—the political economy of the life sciences (Cooper and Waldby, 2014; Biruk, 2018).

## Conclusion

Given the high commercial stakes associated with the research and development, patenting, and approval of monoclonal antibodies and other medical products derived from cell lines—and the necessity of participation by research subjects—informed consent, and all its variations, have been shaped within the commercial interests emerging from the commercialization of products of biomedical research (King and Moulton, 2006; Sharp and Yarborough, 2006; Fisher, 2008; Cooper and Waldby, 2014; Sunder Rajan, 2017). It is with this concern in mind that we tailored our research, aiming to build a framework of research sensitive to the histories and political economy in which these medical projects are situated. The immortalization of cell lines, their subsequent hybridization, the resulting non-stop production of monoclonal antibodies, and their high commercial value successively clash with the ephemeral nature of patients with cancer, whose needs go beyond the values and progressive aims of biomedicine.^10^ The logic behind immortalized cell lines introduced over a century ago, and now underpinning monoclonal antibodies, creates, through their use in oncological clinical trials, an increasing tension between the needs of patients, and the promises to extend patients’ life expectations.

What we propose here is a qualitative analysis of the encounters between epistemic, ethical, and economic values, and their impact on experiences of cancer patients who enter pharmaceutical clinical trials for monoclonal antibodies. We have held that the high stakes of biomedical research, especially those associated with the intellectual property rights to oncological drugs based on monoclonal antibodies, translate into a wide range of missing information in the informed consent process. Indeed, oncological research hints at controversial and complex historical trajectories of biotechnologies and policies underpinning the development of drugs for patients with cancer.^11^ How a better understanding of these issues can help developing the project for a medicine shaped not only by researchers and manufacturers, but also by the medical personnel, patients and their beloved ones, and meant for the wellbeing of all these subjects? We have argued that in order to develop an extended and reflexive understanding of social and ethical aspects of biomedical research, it is helpful to consider both the historical development of monoclonal antibodies and biobanking as biotechnological innovations underpinned by a particular patent regime and specific agendas of governmental and non-governmental organizations, as well as the experiences, expectations, and opinions of donors and workers engaged in biomedical research, that here we have encountered within a qualitative fieldwork. The voices of the medical personnel and those of patients with cancer can disclose the social effects produced by the application of ethical, epistemic and economic norms, to possibly inform qualitative, empirical-based reflections on the functioning of biomedical research, and science at large.

## Data Availability

The original contributions presented in the study are included in the article/Supplementary Material, further inquiries can be directed to the corresponding author.

## Footnotes

1 Since their inception, monoclonal antibodies have been considered anti-tumor agents (cfr. Cambrosio and Keating, 1995).

2 We excluded from our inquiry other relevant but less proximate actors such as insurers, funders, and policymakers.

3 We refer here to “personal data” and “personal information” as synonymous terms since both are the result of social negotiations, with differences that may relate to syntax, production standards, and contexts (cfr. Biruk 2018).

4 Lately, the “dynamic consent” process, which is the reform of methods and concepts at the base of informed consent to facilitate the active engagement of participants in research projects, has been proposed by Jane Kaye and colleagues as a way to ask patients, over time, about their preferences and to obtain their authorization for the management of their samples and data. For instance, this could entail asking if they approve the use of samples for specific projects that might come later, that is, after they have given initial consent. At the same time, through dynamic consent, participants might be informed, *via* web platforms, about relevant outcomes of research, such as aggregate or individual results. The approach proposed through dynamic consent differs from informed consent and other kinds of consent in that participants can decide between different grades of engagement. Patients might then express the wish to give their authorization once and for all, for whatever project and without being contacted anymore, or they might instead choose to require approval for every new use of their data and samples with the possibility of accessing more detailed information.

5 The Witness Seminar has been an institutional regular activity of the Institute of Contemporary British Medicine inaugurated by the Wellcome Trust in 1990, and relocated at UCL and Queen Mary, University of London. The Witness Seminars brought together clinicians, scientists, historians and others interested in contemporary medical history so as to realize the oral history necessary to supplement, or extend, existing records, and to create new resources. The 1993 Witness Seminar on Monoclonal Antibodies to which we refer here, conveyed scientists such as Georges Köhler and César Milstein who conceived the monoclonal antibody biotechnology.

6 Monoclonal antibodies are produced through the assemblage of diverse technologies such as cell immortalization realized during the 1950s, the fusion of human and mouse cells realized in 1965 by Henry Harris and John Watkins, cell refrigeration, and other techniques. The discovery of Milstein and Köhler derives therefore from earlier key technological advancements, (cfr. Cambrosio and Keating, 1995; De Chadarevian, 2002; Landecker, 2007; Radin, 2017).

7 Proprietary information is such when its use implies the payment of loyalties to owners of the associated patent (cfr. Mackenzie et al., 1990).

8 So-called “personalized medicine” or “precision medicine” is rooted in its political economy. Indeed, given that some oncological medicaments for which was granted fast-track approval showed scarce clinical impact and a relevant economic burden for health care systems, both the Food and Drug Administration in 2008 and the European Medicine Agency in 2009 asked manufacturers to reduce impacts unconnected to a clinical utility. The manufacturers answered by developing molecular biomarkers to target prospective respondent patients and to exclude those with a low probability of response so as to boost efficacy and reduce the wastage of resources. (Cfr. D’Abramo and Guastadisegni, 2012).

9 Although we conceive of the social contract as one of the milestones of the social utility of biomedicine, we also hold that an analysis of the conditions and experiences of individuals engaged in the social contract, who are located in specific spatial, temporal, and sociocultural dimensions, should be a constant endeavor to critically verify the validity of this contract. For instance, as highlighted by Kristal Biruk (2018), research fatigue and unfulfilled promises might render the social contract dysfunctional.

10 One of the critiques of clinical trials for monoclonal antibodies, especially those facing a shortened approval process, regards the use of surrogate endpoints, where the biological measures used to temporarily authorize the molecular drug do not necessarily translate into any clinical outcomes, cfr. D’Abramo and Guastadisegni (2012).

11 Although oncology is a field in which significant changes are taking place towards a patient-centered approach (cfr. Holland and Weiss, 2010; Pirl et al., 2014), it remains a realm where the options given to patients are few and clinical trials are often made for drugs that will not be approved. Most cancer drugs tested in clinical trials do not reach the marketplace because they are too toxic or because more effective treatments are already available (cfr. Hay et al., 2014; Wong et al., 2018), while some cancer drugs have been retired from the market after their approval (Fralick et al., 2013; Niraula et al., 2015).
