# Supplementary material for: Biomolecular Prospecting, Informative Gaps, and the Cancer Clinic: A Qualitative Fieldwork and an Epistemological, Historical and Ethical Analysis of Informed Consent for Clinical Trials for Monoclonal Antibodies and Biobank Research
Source: Front Genet. 2022 Jun 13;13:872211. doi: 10.3389/fgene.2022.872211 (PMC9238291; doi:10.3389/fgene.2022.872211)
Supplement: Supplementary file 1 [file Table1.DOCX]

Questionnaire A on clinical trials

______________________________________________________________________

**A0** What is the name of the clinical study you are involved?

**A1** I have been informed how long my participation in this clinical trial is likely to last.

False

Unsure

True

**A2** All the treatments and procedures in my clinical trial are standard for my type of cancer.

False

Unsure

True

**A3** There may not be direct medical benefit to me from my participation in this clinical trial.

False

Unsure

True

**A4** If I have not wanted to participate in this clinical trial, I could have declined to sign the consent form.

False

Unsure

True

**A5** I will have to remain in the clinical trial even if I decide someday that I want to withdraw.

False

Unsure

True

**A6** Who firstly spoke with you about this trial?

1. Treating doctor within the hospital

2. treating doctor out the hospital

3. others, specify

**A7** Did you receive information on alternative treatments or interventions to the trial (chemotherapy, other medications, surgery)?

1. Yes,
2. which kind? ____________
3. No

**A8** Regarding your trial, from what sources you received information?

(You can thick more than one option)

Official documents,

Doctor responsible for the study,

Other doctors on the ward

Study nurse(s)

Internet

Others, please specify ________________

**A9** What person or source of information was most helpful in your decision?

(You can tick more than one option)

Official Documents

Treating doctor

Other doctors

Study nurses

Relatives

Internet

Other__________________________

**A 10** How did this/these person(s) help you in making your decisions?

s/he gave me time to ask questions

gave me detailed information on the study/treatment

trust because her knowledge/expertize

as she makes me comfortable

as she knows what is good for me

others, please specify ___________________

**A11** What was your main motivation in participating in the trial?

(you can thick more than one)

Accessing an effective treatment

Help future patients

Better monitory of your disease (more regular examinations)

Others, please specify _____________________
