# Supplementary material for: Biomolecular Prospecting, Informative Gaps, and the Cancer Clinic: A Qualitative Fieldwork and an Epistemological, Historical and Ethical Analysis of Informed Consent for Clinical Trials for Monoclonal Antibodies and Biobank Research
Source: Front Genet. 2022 Jun 13;13:872211. doi: 10.3389/fgene.2022.872211 (PMC9238291; doi:10.3389/fgene.2022.872211)
Supplement: Supplementary file 2 [file Table2.DOCX]

Questionnaire B on biobank research

______________________________________________________________________

**B1** I have signed an additional consent form to authorize researchers to store my biological samples and to do research on these samples and my personal data.

False

unsure

True

**B2** The research done on my samples will have direct benefit for me.

False

unsure

True

**B3** The research done on my samples/data will be of benefit for future generations.

False

unsure

True

**B4** The aim of the research done on my samples/data is to explore the genetic basis for reactions to prescription drugs.

False

unsure

True

**B5** I could ask clinicians or researchers to delete my personal data from the biobank and destroy my biological samples

False

unsure

True

**B6** Would you like to know what are the results of the research done with your samples:

general results? O yes O no

personal results? O yes O no

**B7** Please, think to the fact that researchers and clinicians may access your personal data (medical records and other personal details of your life). What do you think about it?

**B8** How would you feel if your biological material and personal data would be stored over a long period of time for unknown future research projects?

**B9** Would you like to take active part on the management of your personal data/samples?

Yes, because ____

No, because _____

I don’t know
