# Supplementary material for: Biomolecular Prospecting, Informative Gaps, and the Cancer Clinic: A Qualitative Fieldwork and an Epistemological, Historical and Ethical Analysis of Informed Consent for Clinical Trials for Monoclonal Antibodies and Biobank Research
Source: Front Genet. 2022 Jun 13;13:872211. doi: 10.3389/fgene.2022.872211 (PMC9238291; doi:10.3389/fgene.2022.872211)
Supplement: Supplementary file 3 [file Table3.DOCX]

List of Codes used in Atlas.ti to analyse patients’ interviews

______________________________________________________________________

List of acronyms

**BasicInfo**: Basic information; **BB**: Biobank research; **ISs**: Informative Sheets; **DC**: Dynamic Consent; **DMP**: Decision Making Process; **M2P**: Motivation to Participate; **Mn2P**: Motivation not to Participate; **Q**: (our) Questionnaire; **SocioDem Data**: Sociodemographic data; **Trial Exp**: Patients’ experience of their participation in the clinical trial; **Trial Info**: Information on the trial

**BasicInfo**

BasicInfo Context of the interview

BasicInfo Context of the interview: drugs' side effects

BasicInfo Interviewee's context description

**BB**

BB ISs wasting of time

BB lack of information leading to lack of understanding

BB data and sample use

BB data and sample use: secrecy/transparency

BB data and sample use: questioning use of personal data

BB DC burden due to sickness

BB DC doubts of understanding

BB DC impossibility to understand medical expertise

BB difficulties of understanding

BB DMP lack of information

BB DMP patient doesn’t want to know

BB DMP patient wants to know

BB DMP autonomy

BB DMP dynamic consent: negative

BB DMP dynamic consent: positive

BB DMP risks/benefits evaluation

BB DMP supporting broad consent

BB information source(s)

BB ISs

BB ISs lack of recall

BB M2P personal benefits: therapeutic misconception

BB M2P personal benefits

BB M2P social contract

BB M2P supporting future generations

BB M2P supporting research

BB patient accepted participation

BB patient declined participation

BB patient understood

BB Q negative appreciation

BB Q positive appreciation

BB questioning research

BB research findings: problems of understanding

BB research findings: interested in

BB research findings: not interested in

BB right to withdraw

BB TRUST distrust in clinicians

BB TRUST distrust in the system

**DMP**

DMP difficulties in understanding

DMP have to consent/routinised consent

DMP information overload

DMP lacks of information

DMP medical trial & BB: unrelatedness

DMP patient wants trial medication

DMP resignation

DMP autonomy

DMP commission decides

DMP doctor decides

DMP doubts

DMP helpfulness of cooperating doctors

DMP helpfulness of doctors

DMP helpfulness of family members

DMP helpfulness of ISs

DMP helpfulness of nurses

DMP informed decision making

DMP lack of alternatives

DMP lack of autonomy

DMP lack of decision due to pre-conditions

DMP long time for deciding

DMP opinion of family members

DMP paternalism: positive

DMP paternalism: reprimand

DMP paternalism

DMP patient decides

DMP patient doesn't want to know

DMP patient refuses the trial: blamed from medical personnel

DMP patient want to know

DMP risks/benefits evaluation

DMP short time for deciding

DMP trial or chemotherapy

DMP trust vs. autonomy

DMP unhelpfulness of family members

DMP side effects

DMP time for deciding

**INFORMATION SOURCE**

INFORMATION SOURCE internet

INFORMATION SOURCE internet: negative

INFORMATION SOURCE ISs

INFORMATION SOURCE press

INFORMATION SOURCES doctors

INFORMATION SOURCES doctors: missing info

INFORMATION SOURCES external doctors

INFORMATION SOURCES internet: negative - no info available

INFORMATION SOURCES nurses

**ISs**

ISs contact person

ISs difficulties in understanding

ISs difficulties in understanding: disease inexperience

ISs difficulties in understanding: fear dominates

ISs difficulties in understanding: feelings/fears

ISs difficulties in understanding: lack of expertise

ISs difficulties in understanding: language

ISs difficulties in understanding: legal language use

ISs difficulties in understanding: missing information

ISs difficulties in understanding: too abstract

ISs doubts on completeness of information

ISs general considerations

ISs insurances

ISs lack of recall

ISs negative appreciation: contradictions

ISs negative appreciation: excuses

ISs negative emotions/feelings

ISs positive appreciation: clear

ISs positive appreciation: complete and honest on side effects

ISs positive appreciation: exhaustive

ISs positive appreciation: honest on personal benefits

ISs reading

ISs reading - support from relatives

ISs responsibility

ISs side effects

ISs text length/layout

ISs wasting of time

**M2P**

M2P altruistic reasons

M2P bent/persuaded/convinced by medical personnel

M2P better experience

M2P expectations

M2P heard good things about it

M2P hopes in a more comprehensive care of cancer

M2P maintaining improving quality of life

M2P personal benefits: additional examinations

M2P personal benefits: hope in effectiveness of treatment

M2P personal benefits: life saving

M2P supporting research

M2P to help future generations

M2P to help future generations: daughters

M2P to help future generations: not so much

M2P trial more promising/working better than standard chemo

M2P lack of options

Mn2P not necessary

Mn2P placebo

**Q**

Q feedback

Q methods

Q misunderstanding of Q

Q negative appreciation

Q negative appreciation: language difficulties

Q negative appreciation: Q didn't address practical critical aspects of patient's experience

Q positive appreciation

**SocioDem Data**

SocioDem Data family status

SocioDem Data mother tongue

SocioDem Data name

SocioDem Data age

SocioDem Data children

SocioDem Data gender

SocioDem Data profession

SocioDem Data religion

SocioDem Data time since signature

SocioDem Data work status

SocioDem Data education level

**TRIAL EXP**

TRIAL EXP resignation with experimental nature of the trial

TRIAL EXP negative treatment outcomes

TRIAL EXP personal benefits: additional examinations

TRIAL EXP positive treatment outcomes

TRIAL EXP withdraw from trial

TRIAL EXP acceptance of trial procedures

TRIAL EXP gambling with trial

TRIAL EXP moral & ethics: lack of

TRIAL EXP negative/emotions feelings

TRIAL EXP negative/emotions feelings: communicative delay

TRIAL EXP negative/emotions feelings: disappointment

TRIAL EXP negative/emotions feelings: discomfort with experimental nature of the trial

TRIAL EXP negative/emotions feelings: lack of autonomy

TRIAL EXP negative/emotions feelings: unknown doctor

TRIAL EXP no consideration of person

TRIAL EXP objectification of patient

TRIAL EXP patient wants to know

TRIAL EXP positive/emotions feelings

TRIAL EXP side effects

TRIAL EXP unclear responsibilities

**TRIAL INFO**

TRIAL INFO aims

TRIAL INFO alternative treatment: chemotherapy

TRIAL INFO alternative treatments

TRIAL INFO authorisation: EU/USA

TRIAL INFO biology of the trial

TRIAL INFO compensations

TRIAL INFO current trial status

TRIAL INFO doctors network handles the patient

TRIAL INFO information/encounter with doctors

TRIAL INFO information/encounter with nurses

TRIAL INFO length of the trial/therapy

TRIAL INFO methodology of the trial

TRIAL INFO name

TRIAL INFO patient wants to know

TRIAL INFO personal benefits: unknown

TRIAL INFO positive appreciation: exhaustive

TRIAL INFO positive appreciation: medication

TRIAL INFO side effects

TRIAL INFO study protocol

TRIAL INFO timing

TRIAL INFO uncertainty of information

TRIAL INFO uncertainty of the trial - experimental nature

TRIAL INFO withdrawal from the trial

TRIAL INFO data sharing and data protection

TRIAL INFO participation in previous trial

TRIAL INFO participation is voluntary

TRIAL INFO placebo

**TRIAL PROCEDURE**

TRIAL PROCEDURE negative: communication

TRIAL PROCEDURE negative: mistake

TRIAL PROCEDURE negative: pat does not feel support

TRIAL PROCEDURE negative: refused from trial

TRIAL PROCEDURE negative: unclear organisation (unorganised)

TRIAL PROCEDURE negative: examination mistake

TRIAL PROCEDURE negative: too many examinations

TRIAL PROCEDURE responsibility of managing side effects

TRIAL PROCEDURE role of the medical personnel

TRIAL PROCEDURE patient does not want to know

TRIAL PROCEDURE patient wants to know

TRIAL PROCEDURE examinations

TRIAL PROCEDURES patient’s understanding of inclusion/exclusion criteria

**TRUST**

TRUST distrust in doctors

TRUST distrust in insurances

TRUST distrust in the system

TRUST distrust in the therapy

TRUST in the trial

TRUST in clinicians/researchers

TRUST in clinicians/researchers: because of expertise

TRUST in clinicians/researchers: because of feelings

TRUST in clinicians/researchers: because of treatment
